# Evaluation of pharmaceutical lifesaving skills training oriented pharmaceutical intervention

**DOI:** 10.1186/s40780-016-0054-7

**Published:** 2016-09-07

**Authors:** Yoshito Zamami, Toru Imai, Masaki Imanishi, Kenshi Takechi, Naoko Shiraishi, Toshihiro Koyama, Hidenori Sagara, Yasukazu Shiino, Toshiaki Sendo, Keisuke Ishizawa

**Affiliations:** 1 Department of Clinical Pharmacy, Institute of Biomedical Sciences, Tokushima University Graduate School, 2-50-1 kuramoto-cho, Tokushima, 770-8503 Japan; 2 Department of Pharmacy, Tokushima University Hospital, 2-50-1 kuramoto-cho, Tokushima, 770-8503 Japan; 3 Department of Emergency Pharmaceutical Sciences, Okayama University Graduate School of Medicine, Dentistry and Pharmaceutical Sciences, 2-5-1 Shikata-cho, Kita-ku, Okayama, 700-8530 Japan; 4 Department of Pharmacy, Nihon University Itabashi Hospital, 30-1 Oyaguchi-Kami Machi, Itabashi-ku, Tokyo, 173-8610 Japan; 5 Clinical Trial Center for Developmental Therapeutics, Tokushima University Hospital, 2-50-1 Kuramoto-cho, Tokushima, 770-8503 Japan; 6 Department of Hospital Pharmacy, Okayama University Hospital, 2-5-1 Shikata-cho, Kita-ku, Okayama, 700-8558 Japan; 7 Department of Clinical Pharmacy, Graduate School of Medicine, Dentistry and Pharmaceutical Sciences, Okayama University , 2-5-1 Shikata-cho, Kita-ku, Okayama, 700-8558 Japan; 8 Department of Pharmaceutical Information Sciences, Matsuyama University, 4-2 Bunkyo-cho, Matsuyama, Ehime 790-8578 Japan; 9 Department of Acute Medicine, Kawasaki Medical School, 577 Matushima, Kurahiki, Okayama, 701-0192 Japan

**Keywords:** Pharmaceutical lifesaving skills training, Simulation education, Correspondence structural analysis

## Abstract

**Background:**

Many pharmacists are participating in team-based medical care in emergency hospitals. Therefore, there is a desperate need to improve the education system. In the present study, we provided a “pharmaceutical lifesaving skills training” to the students in their fifth and sixth year of the pharmaceutical school and evaluated the program’s impact on the students’ learning and confidence in their ability to perform pharmaceutical interventions for emergency patients.

**Methods:**

We conducted a pharmaceutical lifesaving skills training program with 12 participants who were in their fifth and six year of pharmaceutical school. We prepared a fictional scenario in which a patient with cardiac arrest has been rushed into a hospital. We measured the participants’ level of knowledge of pharmaceutical lifesaving procedures and participants’ confidence to perform pharmaceutical interventions before and after the training session. Using the data obtained from type II quantification method, we examined what elements in the content of the pharmaceutical lifesaving skill training attended by pharmacy students will affect the students’ confidence to perform pharmaceutical interventions. In addition, using the correspondence structural analysis, we examined which sections of the content of the pharmaceutical lifesaving skill training should be improved in the future.

**Results:**

When we evaluated the level of knowledge acquired in pharmaceutical lifesaving skills training, the post-training overall correct answer rate was significantly higher than the pre-training overall correct answer rate. And also, level of participants' confidence to perform pharmaceutical interventions similarly increased after pharmaceutical lifesaving skill training. The influence degree graph indicates that the items likely to have a major impact on the participants’ confidence to perform pharmaceutical interventions was “Selecting medicine”. According to the correspondence structural analysis graph based on the questionnaire survey, one item identified as an improvement required was “Selecting medicine”.

**Conclusions:**

Our high-performance patient simulator-based lifesaving skills training program not only increased the participants’ understanding of the training content but also increased their confidence in their ability to perform pharmaceutical interventions. Therefore, the pharmaceutical lifesaving skills training program we developed will contribute to the education of emergency care pharmacists who can perform pharmaceutical interventions for emergency patients.

## Background

In recent years, the environment that surrounds a pharmacist has changed dramatically because of increasingly advanced and complex health services. Against this social backdrop, the model or core curriculum in pharmaceutical education was revised in 2013. The curriculum content has been greatly changed. Students are now required to gain knowledge in the areas not clearly stated in the previous curriculum, including emergency care, palliative care, and physical assessment. Until now, the instructions in palliative care [1] and physical assessment [2–4] have been provided in pharmacy schools and the educational impact has been reported, but there has still not been an adequate increase in the instructions in emergency care. However, with the 2008 revision of the medical payment system, the patients requiring emergency admission or special administration for intensive care can now be included in the payment scheme for pharmaceutical services even if communication by language is difficult between the parties. Further, many pharmacists are participating in team-based medical care in emergency hospitals [5–7]. Therefore, there is a desperate need to improve the education system. In order to promote emergency medical care and educate the pharmacists in pharmacology for emergency patients requiring swift diagnosis, we developed and reported the educational impact of life saving skill training given to pharmacy students [8]. This training program makes use of high-performance patient simulator and places emphasis on pharmacology, as well as collecting medication information, choosing the optimum drugs to administer, and preparing medicine. However, we conducted a pilot study with a small number of students. We believe it is necessary to make further improvements to the training program so that it delivers better educational outcomes.

Therefore, we provided a “pharmaceutical lifesaving skills training” to the students in their fifth and sixth year of the pharmaceutical school and evaluated the program’s impact on the students’ learning and confidence in their ability to perform pharmaceutical interventions for emergency patients. We also examined the various aspects of the training content that can be changed in order to improve future training.

## Methods

### Overview of the training program

In April 2013, we conducted a pharmaceutical lifesaving skills training program with 12 participants, five of whom were in their fifth year, and the remaining seven were in their sixth year of department of emergency pharmaceutical sciences, okayama university graduate school of medicine, dentistry and pharmaceutical sciences. We prepared a fictional scenario in which a patient with cardiac arrest has been rushed into a hospital. Based on this scenario, the training focused on five items: (1) using the patient’s medicine envelope/medicine notebook to obtain medication information, (2) choosing the medicine according to the patient’s symptoms, (3) preparing the medicine taking into account the dosage and administration method, (4) setting up an intravenous drip, and (5) common techniques used by medical staff (setting up ECG monitor, chest compression, defibrillation, et cetera). After 30 min of prior presentation for training in the handling of the mannequin, students began to work on the simulated patient along a scenario and performed it for approximately 120 min.

Regarding the allocation of roles, the participants consulted their fellow team members and coordinated their efforts to help in treating the patient. In this way, the training program was designed to give the participants a general grounding in team dynamics. After the training session was completed, the participants reflected on the treatment they had provided to the simulated patient, and then the instructor provided an explanation of the patient’s condition and the appropriate medicines, as well as feedback.

### Evaluating the level of knowledge acquired in pharmaceutical lifesaving skill training

We measured the participants’ level of knowledge of pharmaceutical lifesaving procedures before and after the training session. Specifically, we set the participants pre and post-training tests, both of which involved answering ten true- or- false questions concerning the training content. We used the correct answer rate as a knowledge-acquisition index. The same questions appeared on both tests. The contents of the questions used on the knowledge-acquisition tests are shown in Table 1.

**Table 1:** Knowledge degree test for pharmaceutical life-saving skills training

For the following questions, please mark “O” for True and “X” for False.
1. We give adrenaline every 20–30 minutes. ( )
2. Pharmacotherapy is applied to PEA and asystole, not defibrillation. ( )
3. Magnesium sulfate induces torsades de pointes. ( )
4. Lidocaine has higher heartbeat relapse rates than Amiodarone. ( )
5. We give 0.3 mg of adrenaline for cardiac arrest. ( )
6. When we administer medication to patients with cardiac arrest, we elevate the arm to a position that is higher than the heart for 10–20 seconds after giving 5 mL normal saline. ( )
7. The vasopressin causes vasoconstriction through the V_1_ receptor. ( )
8. Amiodarone has fewer proarrhythmic effects than Nifekalant. ( )
9. Administration of atropine is recommended for PEA and asystole in the 2010 cardiopulmonary resuscitation guidelines. ( )
10. We give sodium bicarbonate for tricyclic antidepressant poisoning. ( )

### Evaluation of training content provided in pharmaceutical lifesaving skill training questionnaire

We administered a questionnaire survey to the participants in order to measure the educational outcomes of the lifesaving skills training program. We set questions that inquired into the participants’ level of understanding of the training content. We also set questions that inquired into the participants’ confidence in their ability to perform pharmaceutical interventions. We conducted the questionnaire survey before and after the training. We ranked each question on a four-point scale, ranging from 1 to 4. Table 2 shows the content of the questionnaire survey on pharmaceutical lifesaving skill training. We prepared the survey on the assumption that Question 5 would be the response variable and Questions 1–4 would be explanatory variables. We also subjected the questionnaire results to the type II quantification method and a correspondence structural analysis. Using the data obtained from type II quantification method, we examined what elements in the content of the pharmaceutical lifesaving skill training attended by fifth-and-sixth-year pharmacy students will affect the students’ confidence in their ability to perform pharmaceutical interventions. The participant’s confidence was set as an objective variable and the understanding of each practice item was set as an explanatory variable. The range score was used to evaluate the extraction of an important factor, which is the strength of the effect on the objective variable [9]. In addition, using the correspondence structural analysis, we examined which sections of the content of the pharmaceutical lifesaving skills training should be improved in the future. We used Excel Statistical Quality Control (ESUMI Co., Ltd.) as the statistical processing software for the correspondence structural analysis.

**Table 2:** Pharmaceutical life-saving skills training questionnaire

Before training
1. Can you perform pharmaceutical interventions for real cardiopulmonary arrest patients?
(Does not apply at all) **1 – 2 – 3 – 4** (Applies completely)
After training
In today’s training, did you understand how to select the medicine?
(Did not understand at all) **1 – 2 – 3 – 4** (Understood completely)
2. In today’s training, did you understand how to gather information on drugs taken?
(Did not understand at all) **1 – 2 – 3 – 4** (Understood completely)
3. In today’s training, did you understand how to prepare the medicine?
(Did not understand at all) **1 – 2 – 3 – 4** (Understood completely)
4. In today’s training, did you understand how to select the medicine?
(Did not understand at all) **1 – 2 – 3 – 4** (Understood completely)
5. Can you perform pharmaceutical interventions for real cardiopulmonary arrest patients?
(Does not apply at all) **1 – 2 – 3 – 4** (Applies completely)

### Statistical analysis

We compared the overall correct answer rates using a paired t-test. We conducted a statistical analysis using *p* < 0.05 as the significance threshold, and we plotted the correct answer rate (%) ± standard error on a graph.

## Results

**Fig. 1:**
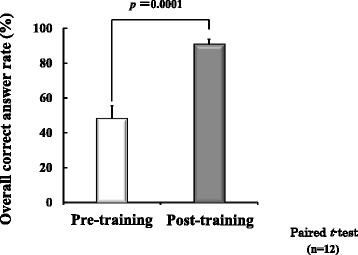
Evaluation of knowledge acquisition of training content

**Fig. 2:**
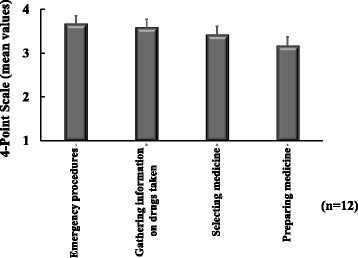
Level of understanding of training content

**Fig. 3:**
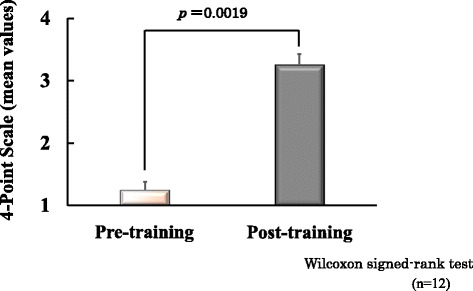
Level of participants’ confidence to perform pharmaceutical interventions

When we evaluated the level of knowledge acquired in pharmaceutical lifesaving skills training, we found that the post-training overall correct answer rate (83.8 ± 1.7) was significantly higher than the pre-training overall correct answer rate (52.2 ± 2.4); see Fig. 1. Figure 2 shows the post-training results indicating the level of understanding of the training content. The level of understanding of the training content was as follows: Emergency measures = 3.8 ± 0.1, Gathering information on drugs taken =3.6 ± 0.2, Preparing medicine =3.4 ± 0.2, selecting medicine = 3.1 ± 0.2. Level of participants' confidence to perform pharmaceutical interventions significantly increased after pharmaceutical lifesaving skill training (Fig. 3).

**Fig. 4:**
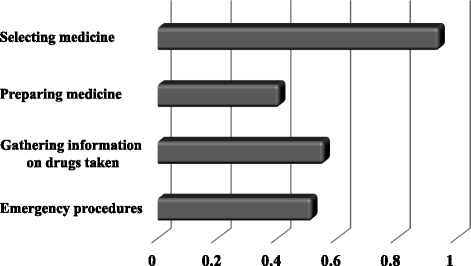
Influence degree graph in Type 2 quantification

**Fig. 5:**
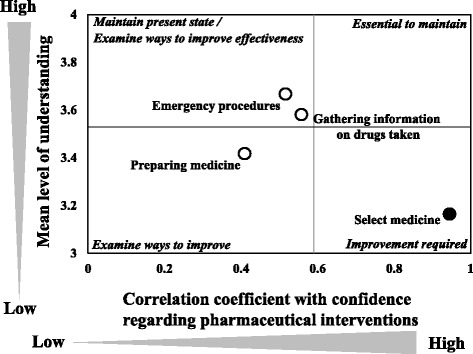
Customer satisfaction analysis of training content evaluation

We plotted influence degree graph that shows the evaluation of the training content using the type II quantification method (Fig. 4). The graph indicates that the items likely to have a major impact on the participants’ confidence in their ability to perform pharmaceutical interventions. Figure 5 is a correspondence structural analysis graph for the evaluation of training items based on the questionnaire survey. According to the results, one item identified as an improvement required item was “Selecting medicine”: (In today’s training, did you understand how to select the medicine?).

## Discussion

We implemented and evaluated the educational efficacy of the lifesaving-training program for pharmacy students. The program made use of a high-performance patient simulator, which simulated a patient with cardiac arrest being rushed into a hospital outpatient care, and it placed emphasis on the pharmaceutical interventions. Our evaluation of the program indicated that it deepens the participants’ understanding of emergency measures, including pharmaceutical measures, and that it increases their confidence in their ability to perform pharmaceutical interventions. The results also yielded a new finding that item of “selection of medicine should be changed in order to improve the training content.

Emergency care involves a distinctive form of diagnostic care of emergency and serious patients, which differs from general diagnostic care. Emergency care is a relatively recent discipline in Japan, and many pharmacists are not adequately aware of its distinctive nature. This situation must be improved as soon as possible. Hence, there is an urgent need to enhance and spread education on clinical emergency care among pharmacists. An effective educational strategy is off-job simulation training for emergency care. In this study, we tested the participants’ level of acquired knowledge of the content of the lifesaving skills training in order to measure the learning outcomes of our pharmaceutical lifesaving skill-training program. The results indicated that the post-training overall correct answer rates were significantly higher than the pre-training overall correct answer rates. Moreover, in the post-training questionnaire survey, the participants showed a high level of understanding of all items (emergency measures gathering information on medicine taken, preparing injection/intravenous drip, and selecting medicine). It has been reported that when teaching CPR to medical students, high-performance patient simulators provide a greater sense of realism than traditional simulators and deliver better learning outcomes [10]. In addition, we prepared a scenario based on the type of cases experienced daily by pharmacists employed in emergency care settings. Therefore, we believe that this scenario reflected real-life clinical practice and led to better learning outcomes.

The participants’ confidence in their ability to perform pharmaceutical interventions to emergency patients was markedly higher after the training. The fact that the training increased the participants’ confidence in their ability to perform pharmaceutical interventions, should have a positive impact on their proactivity, which is indispensable for promoting the involvement of pharmacists in outpatient emergency care in the future. The sense of accomplishment felt by the students when they succeeded in restarting the high-performance patient simulator’s heartbeat after a collaborative process of trial and error probably caused a major boost in their self-confidence.

The item that had the greatest impact on the participant’s confidence in their ability to perform pharmaceutical interventions was probably “selecting medicine”. One probable cause of this result is the way we designed the training. That is, we gave the participants enough time to consult among themselves while referring to the available sources of information (such as the notes attached to the drugs and emergency treatment guidelines) that would help them figure out how to treat the case. We also set up the simulator patient so that it would die if an inappropriate procedure were performed. In other words, the participants probably recognized that an understanding of how each drug should be used in emergency cases would be useful when performing pharmaceutical interventions for actual emergency patients. Doctors and nurses in emergency outpatient care must administer drugs while performing various emergency procedures, so it is difficult in many cases to select the optimum medicine for the patient’s condition. Therefore, the students who participated in our lifesaving skills training program will improve the quality of their pharmacotherapy skills with respect to the selection of medicine for emergency outpatients, and thereby promote proper use of drugs and the prevention of excessive administration.

Subsequently, we analyzed the training content we provided using a Customer Satisfaction (CS) analysis in order to find out how we can improve the training in the future. According to the CS analysis, the item most in need of improvement is “selecting medicine”. On the CS graph, this item was positioned in the improvement required category, denoting that the item was poorly understood and negatively affected the overall evaluation. The training did not provide the participants with knowledge on which medical treatments will have a beneficial effect on emergency patients. It was the pharmacy students’ first time selecting drugs to administer to the emergency patient, so their lack of familiarity with the process may have caused this result. This aspect should be improved by familiarizing the participants as much as possible during the training. For example, the participants can be given a lecture on the CPR-related drugs that are covered in the training. In light of the results of the CS analysis, we concluded that the content of “Selecting medicine” should be changed in order to improve the pharmaceutical lifesaving skills training program.

A limitation of this study is the lack of participation by doctors and nurses, who play a vital role in the delivery of emergency procedures. It cannot be stated that the universal efficacy of the program has been shown completely by only analyzing the evaluation of the small number of participants. Using the present pharmaceutical lifesaving skill training program as a reference, we would like to develop a practical pharmaceutical lifesaving skill training program based on interdisciplinary collaboration.

## Conclusion

Our high-performance patient simulator-based lifesaving skills training program not only increased the participants’ understanding of the training content but also increased their confidence in their ability to perform pharmaceutical interventions. Therefore, the pharmaceutical lifesaving skills training program we developed will contribute to the education of emergency care pharmacists who can perform pharmaceutical interventions for emergency patients.

We expect that the pharmacy students who attended our pharmaceutical lifesaving skill training will be posted in various emergency care facilities where they will help save many patients’ lives.

The contents of this article won an excellent subject prize in the 24th annual meeting of the Japanese society of pharmaceutical health care and sciences (In September, 2014, Nagoya).
